# Comparison of the Effects of Continuous Subcutaneous Insulin Infusion and Add-On Therapy with Sitagliptin in Patients with Newly Diagnosed Type 2 Diabetes Mellitus

**DOI:** 10.1155/2016/9849328

**Published:** 2015-12-21

**Authors:** Heng Wan, Defu Zhao, Jie Shen, Lu Lu, Tong Zhang, Zhi Chen

**Affiliations:** ^1^Department of Endocrinology and Metabolism, The Third Affiliated Hospital of Southern Medical University, Guangzhou 510630, China; ^2^School of Traditional Chinese Medicine, Southern Medical University, Guangzhou 510515, China

## Abstract

To identify a new regimen to optimize treatment for patients with newly diagnosed type 2 diabetes (T2DM) by short-term continuous subcutaneous insulin infusion (CSII) alone.* Methods. *60 patients with newly diagnosed T2DM were randomized into two groups (*n* = 30 each) and treated for 2 weeks with CSII alone (CSII group) or with CSII plus sitagliptin (CSII + Sig group). The glycemic variability of the patients was measured using a continuous glucose monitoring system (CGMS) for the last 72 hours. A standard meal test was performed before and after the interventions, and the levels of glycated albumin, fasting glucose, fasting C-peptide, postprandial 2 h blood glucose, and postprandial 2 h C-peptide were examined.* Results.* Compared with the CSII group, the indicators of glycemic variability, such as the mean amplitude of glycemic excursion (MAGE) and the standard deviation of blood glucose (SDBG), were decreased significantly in the CSII + Sig group. The changes before and after treatment in the C-peptide reactivity index (ΔCPI) and the secretory unit of islet in transplantation index (ΔSUIT) indicated a significant improvement in the CSII + Sig group.* Conclusions.* Add-on therapy with sitagliptin may be an optimized treatment for patients with newly diagnosed T2DM compared with short-term CSII alone.

## 1. Introduction

Type 2 diabetes mellitus (T2DM) is a chronic disease that is characterized by progressive *β*-cell dysfunction that leads to insulin deficiency. At the time of diagnosis, *β*-cell function may be reduced by as much as 50% compared with healthy control subjects and will progressively deteriorate over time, irrespective of lifestyle and pharmacological interventions, as revealed in the UK Prospective Diabetes Study [1]. Recent studies have suggested that short-term intensive insulin therapy, with continuous subcutaneous insulin infusion (CSII) potentially being the best current therapeutic option [2, 3], can rapidly relieve newly diagnosed T2DM patients of high glucose toxicity, ameliorate the state of insulin resistance, and restore islet *β*-cell function [4, 5]. However, glycemic variability and hypoglycemia are risks associated with this type of therapy [6, 7].

Studies suggest that glycemic variability, an HbA1c-independent risk factor, is another important factor leading to chronic complications of diabetes [8–10]. Some studies have even suggested that glycemic variability has more deleterious effects than sustained hyperglycemia in the development of diabetic complications [11, 12]. Therefore, glycemic variability should be one of the criteria for evaluating glycemic control.

A continuous glucose monitoring system (CGMS) can provide information concerning glucose concentrations throughout the day by monitoring levels every 5 min, thus helping to detect trends in glycemic variability, hyperglycemia, and hypoglycemia that are more difficult to detect using conventional self-monitoring of blood glucose [13].

Our previous study [14] showed that the area under the curve of glucagon-like peptide-l (GLP-1) in patients with T2DM was significantly lower than that in patients with normal glucose tolerance or impaired glucose tolerance. However, the dipeptidyl peptidase-4 (DPP-4) inhibitor sitagliptin can increase active GLP-1 concentrations and thereby enhance insulin secretion by *β*-cells and inhibit glucagon release from *α*-cells in a glucose-dependent manner [15]. Therefore, our present study uses CGMS to evaluate the impact of glycemic variability when adding sitagliptin to the CSII therapy of newly diagnosed T2DM patients.

In addition, previous studies have suggested that sitagliptin can restore *α*-cell function, ameliorate insulin resistance (HOMA-IR), and improve pancreatic *β*-cell function (HOMA-*β*) in patients [16]. In our study, sitagliptin is added to short-term CSII to control the glucose of newly diagnosed T2DM patients to determine whether there is an improvement in pancreatic *β*-cell function.

The aim of this randomized controlled trial is to compare the effects of CSII alone with those of CSII combined with sitagliptin in newly diagnosed T2DM patients in an attempt to optimize a therapeutic regimen for such patients.

## 2. Methods

### 2.1. Subjects

Sixty inpatients newly diagnosed with T2DM according to the 1999 World Health Organization diagnostic criteria were recruited in The Third Affiliated Hospital, Southern Medical University, Guangzhou, China, from September 2014 to May 2015. The following inclusion criteria were used: (1) age between 30 and 70 years, fasting plasma glucose (FPG) between 8 and 16.7 mmol/L, glycosylated hemoglobin (HbA1C) ≥8.0%, and a body mass index (BMI) between 18 and 28 kg/m^2^; (2) negative for glutamic acid decarboxylase autoantibody (GAD-Ab), anti-islet cell autoantibody (ICA-Ab), and anti-insulin autoantibody (IAA-Ab); and (3) no previous treatment with an antidiabetic or antihyperlipidemic medication. The following exclusion criteria were used: (1) type 1 diabetes, gestational diabetes, or diabetes with an identifiable secondary cause; (2) significant renal impairment (estimated creatinine clearance <50 mL/min) or elevated alanine or aspartate aminotransferase (ALT or AST, resp.); (3) occurrence of any severe diabetic complications or severe infection in the previous 3 months; and (4) scheduled surgery or serious trauma. Other patients whom the investigator judged to be inappropriate for the study were also excluded.

### 2.2. Study Design and Treatment

The eligible patients were randomized (1 : 1) using a random number table into treatment groups receiving either CSII alone (A: CSII group) or CSII combined with 100 mg sitagliptin once daily (B: CSII + Sig group) for 2 weeks. All patients were treated with insulin aspart (Novo Nordisk, Bagsvaerd, Denmark) using insulin pumps (MiniMed 712E, Medtronic, Northridge, CA, USA). The initial daily insulin dosage was calculated as follows: total insulin dose daily = 0.5 unit × body weight (kg). The basal rate (units/h) was calculated as 50% of the total insulin dose, and the other 50% was administered as a preprandial bolus before each of the three daily meals. The initial basal dose was divided into six doses that were administered during the following six periods of the day: 00:00 to 03:00 h, 03:00 to 07:00 h, 07:00 to 12:00 h, 12:00 to 17:00 h, 17:00 to 22:00 h, and 22:00 to 24:00 h. Capillary blood glucose was monitored eight times per day (before and 2 h after each meal, at bedtime, and at 03:00 h). The basal and bolus doses of insulin infusion were adjusted daily by one doctor by 2 to 10 units according to the capillary blood glucose level to achieve euglycemia (fasting blood glucose <7.0 mmol/L and postprandial blood glucose <10.0 mmol/L). The CSII was suspended after 2 weeks of treatment, and glucose levels were monitored by CGMS (Medtronic MiniMed, Northridge, CA, USA) during the last three days (72 h) of treatment.

A standard meal tolerance test using a meal consisting of 2037 kcal, 54.8 g of carbohydrate, 25.8 g of fat, and 9.4 g of protein was administered before and after the suspension of CSII treatment. Blood was collected at 0 and 120 min after the meal start. No antihyperlipidemic agents were used during the intervention.

All of the subjects underwent an education program on diabetes self-management, including diet and exercise counseling. A diabetic diet consisting of 50% carbohydrate (200 g), 35% fat, and 15% protein was provided for all subjects during intervention. The distribution of caloric intake was 20% for breakfast, 40% for lunch, and 40% for dinner. Regular physical exercise, such as walking, jogging, or stair climbing, for 30 min after meals was recommended.

This work was approved by the Medical Research and Ethics Committee of the Third Affiliated Hospital of Southern Medical University (Guangzhou, People's Republic of China) and registered at chictr.org (Chinese Clinical Trial Registry) with trial registration identifier number ChiCTR-TRC-14005224. An informed consent was obtained from all participants in this study.

### 2.3. Measurements

Anthropometric and laboratory data, including height, weight, age, BMI, FPG, 2 h postprandial plasma glucose (PPG), glycated hemoglobin (HbA1c), glycated albumin (GA), fasting C-peptide (FC-P), 2 h postprandial C-peptide (PC-P), triglycerides (Tg), total cholesterol (TC), high-density lipoprotein-cholesterol (HDL-C), and low-density lipoprotein-cholesterol (LDL-C), were measured before and after CSII treatment with the exception of HbA1c, which was not measured after treatment. The secretory unit of islet in transplantation (SUIT) index and C-peptide reactivity index (CPI) were used to estimate *β*-cell function. The following formulas were used: SUIT index = 250 × FC-P (ng/mL)/(FPG (mg/dL) − 3.43); CPI = FC-P (ng/mL)/FPG (mg/dL) × 100.

The mean amplitude of glycemic excursions (MAGE), standard deviation of blood glucose levels (SDBG), largest amplitude of glycemic excursions (LAGE), mean blood glucose level (MBG), proportion (%) of time in hyperglycemia (>10 mmol/L) (PT10.0), proportion (%) of time in hypoglycemia (<3.9 mmol/L) (PT3.9), 1 h fasting MBG, and 3 h postprandial MBG were obtained by CGMS.

The difference in insulin dosage (Δinsulin) and the change in body mass index (ΔBMI) before and after treatment were calculated, and the number of hours in which the target blood glucose was reached was recorded.

### 2.4. Statistical Methods

All statistical analyses were performed using SPSS 13.0 software (SPSS, Inc., Chicago, IL, USA). The variables were then examined independently and subjected to normality and homogeneity of variance tests. The data for normally distributed variables are reported as the means ± SD, and the data nonnormally distributed variables are reported as medians and interquartile ranges. Count data are expressed as rates. The independent-samples *t*-test was used to test for differences between two groups. The paired *t*-test was used to test for differences before and after the intervention. Fisher's exact test was used to analyze enumeration data. A two-sided value of *P* < 0.05 was considered statistically significant.

## 3. Results

### 3.1. Baseline Characteristics and General Treatment Efficacy

Sixty patients with newly diagnosed T2DM were recruited for the study and randomized into two groups. No patients dropped out, and no serious adverse effects were observed during the intervention. The baseline features and clinical characteristics of the patients (age, gender, weight, and BMI) were similar between the two groups (*P* > 0.05, Table 1). There were also no significant differences between the groups in glucose levels (HbA1c, GA, FPG, and PPG), lipid profile (TC, HDL-C, LDL-C, and Tg), or indices of *β*-cell secretion (CPI, SUIT) at the beginning of the study (*P* > 0.05, Table 1).

### 3.2. Comparison of Glycemic Excursions

The values of the indices of glycemic excursions derived from the CGMS during the last three days of treatment are shown in Table 2. The LAGE, MAGE, and SDBG of the patients in the CSII + Sig group were all significantly lower than those of the CSII group after the intervention (*P* < 0.01). Similarly, the preprandial 1 h MBG level, the postprandial 3 h MBG level, and the MBG level during the last 72 h of treatment were significantly lower in the CSII + Sig group than in the CSII group (*P* < 0.05). During treatment, the incidence of patients who experienced hypoglycemia (PG < 3.9 mmol/L), as indicated by self-monitoring of blood glucose (SMBG), in the CSII + Sig group was 16.7% (5/30), which was lower than that observed in the CSII group (23.3%; 7/30). However, this difference was not significant (*P* > 0.05). Furthermore, CGMS showed that the PT3.9 of the CSII + Sig group was significantly lower than that of the CSII group (*P* = 0.04). In addition, the PT10.0 of the CSII + Sig group was significantly lower than that of the CSII group (*P* < 0.01).

### 3.3. Changes in GA

After 2 weeks of treatment, the GA reduction from baseline in the CSII + Sig group was 8.02% (final mean GA, 26.09%), and the reduction in the CSII group was 4.82% (final mean GA, 28.60%); both of these changes were significant (*P* < 0.01). The reduction in the GA (ΔGA) in the CSII + Sig group was significantly greater than the reduction in the CSII group (*P* < 0.01, Table 3).

### 3.4. Effects on Glucose Level and *β*-Cell Function

All of the patients in the two groups achieved euglycemia within the two weeks of intervention. However, in both the CSII + Sig and CSII groups, significant reductions from baseline in FPG (5.84 ± 1.05 and 6.55 ± 0.93, resp.) and PPG (12.07 ± 2.79 and 13.77 ± 1.92, resp.) were observed after treatment (*P* < 0.01 and *P* < 0.01, resp.). In addition, the values of SUIT (4.29 ± 1.47 and 3.90 ± 1.39, resp.), CPI (1.66 ± 0.56 and 1.51 ± 0.53, resp.), and PC-P (5.97 ± 2.55 and 5.45 ± 2.40, resp.) were significantly elevated from baseline in both the CSII + Sig and CSII groups (*P* < 0.01, *P* < 0.01, and *P* < 0.01, resp.). The FC-P (1.69 ± 0.51) of the CSII + Sig group increased significantly (*P* = 0.04) after treatment, whereas the FC-P (1.73 ± 0.49) of the CSII group was comparable to baseline (*P* > 0.05).

The changes in *β*-cell function between the two groups were also compared. The increases in SUIT and CPI (ΔSUIT and ΔCPI) from baseline to after treatment were greater in the CSII + Sig group than in the CSII group (*P* = 0.03 and *P* = 0.03, resp., Table 3). The decrease in PPG (ΔPPG) was greater in the CSII + Sig group than in the CSII group (*P* < 0.01, Table 3), whereas the decrease in FPG (ΔFPG) and the increases in C-P and PC-P (ΔC-P and ΔPC-P) did not differ between the groups (all *P* > 0.05, Table 3).

### 3.5. Influence of the Lipid Profile

**Figure 1:**
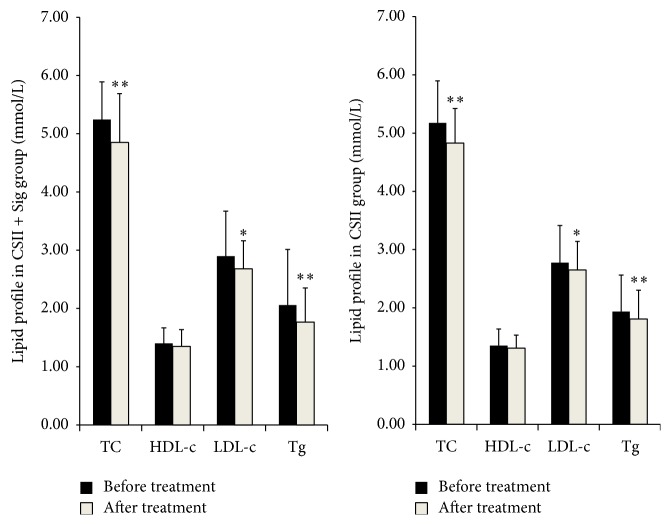
Changes in lipid profile before and after treatment for both groups. Note: ^*∗*^ *P* < 0.05 and ^*∗∗*^ *P* < 0.01; paired *t*-tests (after versus before treatment) were conducted. TC: total cholesterol; HDL-c: high-density lipoprotein-cholesterol; LDL-c: low-density lipoprotein-cholesterol; Tg: triglycerides.

The lipid profile improved to some extent in both groups. Significant suppression of the fasting TC, LDL-c, and Tg levels was observed in both the CSII (*P* < 0.01, *P* = 0.03, and *P* < 0.01, resp., Figure 1) and CSII + Sig (*P* < 0.01, *P* = 0.03, and *P* < 0.01, resp., Figure 1) groups compared with baseline, whereas the change in HDL-c from baseline to after treatment was not significant in either group (both *P* > 0.05, Figure 1). However, the reductions in the fasting lipid profile (ΔTC, ΔLDL-c, ΔHDL-c, and ΔTg) were comparable between the two groups (all *P* > 0.05, Table 3).

### 3.6. Evaluation of Time to Achieve Euglycemia, Dosage of Insulin, and Weight

As shown in Table 3, there was a significantly shorter time to achieve euglycemia and a significant decline in the daily insulin dosage (Δdosage of insulin) in the CSII + Sig group compared with the CSII group (*P* < 0.01 and *P* < 0.01, resp., Table 3). The decline in the bolus insulin dose (Δbolus insulin dose) was significantly greater in the CSII + Sig group than in the CSII group (*P* < 0.01, Table 3), but the changes in the basal insulin dose (Δbasal insulin dose) and the basal/bolus ratio (Δbasal/bolus ratio) were comparable between the groups (*P* > 0.05 and *P* > 0.05, resp., Table 3). No differences were found in the changes in body weight and BMI (Δweight and ΔBMI) between the two groups (both *P* > 0.05, Table 3).

## 4. Discussion

This study assessed the clinical efficacy of sitagliptin in patients with newly diagnosed T2DM receiving CSII treatment, including the reduction in glucose excursion and differences in glucose amelioration, *β*-cell function, and lipid metabolism.

Based on the overview of the available evidence provided by Nalysnyk et al., it appears that glucose variability, characterized by extreme glucose excursions, could be a predictor of diabetic complications, independent of HbA1c levels, in patients with T2DM [17]. Glucose variability also has been shown to be associated with the activation of oxidative stress and the innate immune system, which increases the risk of diabetic complications [18]. Previous studies have suggested that add-on sitagliptin therapy is significantly well tolerated and improves HbA1c, fasting blood glucose, and postprandial blood glucose compared with placebo in T2DM [19–23]. One study suggested that sitagliptin added to CSII treatment decreases glucose variability, such as MAGE [23]. However, in that study, glucose variation was calculated by measuring capillary blood glucose rather than through CGMS; this is a limitation because intermittent glucose monitoring only allows the variation to be estimated. Here, we assessed glucose variability by CGMS and found that the addition of sitagliptin to CSII therapy produced significant reductions in MAGE, LAGE, and SDBG, indicating that add-on sitagliptin therapy can improve glucose variability in patients with newly diagnosed T2DM who receive CSII treatment. This improvement may be attributed to dipeptidyl peptidase-4 inhibitors, which are reported to inhibit the degradation of the endogenous incretin hormones glucagon-like peptide-1 and gastric inhibitory polypeptide, which in turn glucose-dependently promote insulin secretion and inhibit glucagon secretion, thus helping to correct hyperglycemic states [24]. In this regard, our results also suggest that the risk of hypoglycemia may be reduced by add-on sitagliptin therapy as the PT3.9 was decreased in the CSII + Sig group.

Although an improvement in glycemic control using the combination of a DPP-4 inhibitor and insulin was demonstrated in previous studies [19–21], the intervention time in those studies was longer than 3 months. In patients with T2DM receiving CSII with add-on sitagliptin for 2 weeks, HbA1c was also significantly decreased compared with that in patients receiving CSII alone in a study by Yuan et al. [22] However, HbA1c is an indicator of glycemic control over a 3-month period. In our study, GA was used to monitor the glycemic control state, as it is an indicator of glycation, over a 2-3 week period in diabetic patients. Our finding that GA had a greater improvement in the CSII + Sig group than in the CSII group is consistent with previous studies. Because it is influenced by the improvement of postprandial blood glucose, GA is a better indicator of glucose excursion than HbA1c [25]. Our study showed that the 1 h MBG before meals, 3 h MBG after meals, and glucose excursion were ameliorated when sitagliptin was added to the CSII treatment. The greater improvement in glycemic control in patients receiving CSII with sitagliptin may help to restore islet *β*-cell function more effectively.

As glucotoxicity is corrected rapidly, *β*-cell function can be improved in newly diagnosed T2DM patients treated with short-term intensive insulin therapy [5]. Treatment with sitagliptin has also been shown to improve measures of *β*-cell function [16, 26]. Moreover, in previous clinical studies using concomitant therapy of insulin and sitagliptin, the effect of add-on sitagliptin on improving *β*-cell function was investigated in long-term interventions of at least 12 weeks [19–21]. In a study by Yuan et al. [22], the levels of insulin and C-peptide were strikingly increased and HOMA-*β* (the homeostasis model assessment of *β*-cell function) was improved in the CSII plus sitagliptin group compared with the CSII group. In our study, SUIT and CPI were used to assess *β*-cell responsiveness. Similar to the results of Yuan et al.'s study [22], the indicators of *β*-cell function, SUIT and CPI, were increased. These improvements were most likely due to the improvement of glycemic control in the CSII + Sig group compared with the CSII group. Therefore, the benefits of sitagliptin on *β*-cell function cannot be ignored.

As noted in the position statements of the American Diabetes Association and the European Association for the Study of Diabetes (ADA/EASD), the problems of weight and economics should be considered in the management of hyperglycemia in T2DM. Sitagliptin has been shown to be effective and well tolerated in various treatment regimens with a neutral effect on body weight, possibly because sitagliptin treatment augments GLP-1 levels [27]. Additionally, previous studies have indicated that add-on therapy of sitagliptin to various insulin regimens (not including CSII) could decrease daily insulin doses and improve glycemic control without severe hypoglycemia or weight gain [19–21]. With insulin therapy, further improvement is needed for a glycemic control that will not increase hypoglycemia and weight gain to limit the insulin dose when high doses are needed. In our study, there were great improvements in the state of glucose with an insulin dose decrease and without weight gain (sitagliptin in combination with CSII therapy group versus CSII monotherapy group). It is worth noting that significantly fewer days were required to achieve euglycemia when sitagliptin was added to CSII in the present study, which suggests that the combined therapy may shorten hospital stays and reduce hospitalization expenses for T2DM patients. Therefore, add-on therapy with sitagliptin added to CSII can be cost effective by decreasing the insulin dose and shortening the time to achieve euglycemia.

An unfavorable effect on lipids was thought to be one of the potentially modifiable risk factors for coronary artery disease in patients with T2DM according to the UK Prospective Diabetes Study (UKPDS) [28]. As indicated in this and previous studies, lipotoxicity can be eliminated by short-term CSII-based intensive treatment. In our study, the lipid profiles of both groups after 2 weeks of intensive treatment were significantly improved compared with those before treatment as both treatments lowered TC, LDL-c, and Tg levels. However, the favorable effect on lipids was comparable between CSII alone and CSII combined with sitagliptin. Possible explanations for this finding include the following: (1) the length of our study was only 2 weeks; (2) a previous study showed that postprandial plasma levels of TG-rich lipoproteins were reduced after treatment with sitagliptin for 6 weeks [29], whereas only fasting plasma lipids were measured in our study; and (3) there were an insufficient number of samples in our study.

In summary, our randomized trial in which sitagliptin was added to a CSII-based short-term intensive treatment in patients with newly diagnosed T2DM was very effective in improving glycemic excursions, glucose levels, GA, and *β*-cell function; additionally, a reduced incidence of hypoglycemia, a shorter time to achieve euglycemia, a significant reduction in the insulin dosage, and no weight gain were observed compared with CSII alone. CSII plus sitagliptin appears to be a beneficial regimen, particularly for individuals with newly diagnosed T2DM, and it should be tested in a larger, long-term clinical trial.

## Figures and Tables

**Table 1:** Baseline comparisons between the CSII and CSII + Sig groups before treatment.

Characteristic	Group		*P* value
	CSII	CSII + Sig	
Patients	30	30	—
Gender (F/M)	15/15	16/14	—
Age (years)	45.18 ± 7.10	46.40 ± 5.14	0.49
Weight (kg)	63.14 ± 6.45	65.99 ± 9.90	0.19
BMI (kg/m^2^)	23.56 ± 1.48	23.76 ± 3.01	0.74
HbA1c (%)	10.06 ± 1.82	10.47 ± 1.33	0.32
GA (%)	33.42 ± 4.68	34.11 ± 3.86	0.53
FPG (mmol/L)	10.39 ± 0.96	10.23 ± 0.92	0.50
PPG (mmol/L)	17.97 ± 1.48	18.52 ± 1.45	0.15
FC-P (ng/mL)	1.65 ± 0.59	1.48 ± 0.71	0.32
PC-P (ng/mL)	3.99 ± 2.14	4.20 ± 1.70	0.67
CPI	0.89 ± 0.33	0.82 ± 0.42	0.49
SUIT	2.26 ± 0.84	2.09 ± 1.07	0.49
TC (mmol/L)	5.17 ± 0.72	5.25 ± 0.65	0.69
HDL-C (mmol/L)	1.35 ± 0.29	1.40 ± 0.27	0.50
LDL-C (mmol/L)	2.78 ± 0.64	2.90 ± 0.77	0.51
Tg (mmol/L)	1.94 ± 0.62	2.06 ± 0.96	0.57

Note: data are presented as the means ± SD. CSII group: CSII monotherapy group; CSII + Sig group: CSII therapy in combination with sitagliptin group; GA: glycated albumin; FPG: fasting plasma glucose; PPG: postprandial plasma glucose; FC-P: fasting C-peptide; PC-P: 2-h postprandial C-peptide; CPI: C-peptide reactivity index; SUIT: secretory unit of islet in transplantation index; TC: total cholesterol; Tg: triglycerides; HDL-C: high-density lipoprotein-cholesterol; LDL-C: low-density lipoprotein-cholesterol.

**Table 2:** Glycemic variability between the CSII and CSII + Sig groups after the suspension of continuous subcutaneous insulin infusion.

Characteristic	Group		*P* value
	CSII	CSII + Sig	
MAGE (mmol/L)	3.98 ± 0.55	2.84 ± 0.92	<0.01
SDBG (mmol/L)	2.01 ± 0.37	1.42 ± 0.37	<0.01
LAGE (mmol/L)	6.81 ± 1.29	5.55 ± 1.27	<0.01
MBG (mmol/L)	7.95 ± 0.57	6.64 ± 0.37	<0.01
PT10.0 (%)	6.25 ± 1.48	1.50 ± 1.83	<0.01
PT3.9 (%)	2.42 ± 3.60	0.29 ± 0.73	0.04
1 h MBG			
Before breakfast (mmol/L)	6.42 ± 0.45	6.04 ± 0.68	0.01
Before lunch (mmol/L)	6.68 ± 1.03	6.16 ± 0.66	0.02
Before supper (mmol/L)	6.71 ± 0.89	6.17 ± 0.56	0.01
3 h MBG			
After breakfast (mmol/L)	9.78 ± 1.60	7.51 ± 0.64	<0.01
After lunch (mmol/L)	8.78 ± 1.49	7.59 ± 0.56	<0.01
After supper (mmol/L)	9.29 ± 1.78	7.50 ± 0.61	<0.01

Note: MAGE: mean amplitude of glycemic excursions; SDBG: standard deviation of blood glucose; LAGE: largest amplitude of glycemic excursions; MBG: mean blood glucose; PT3.9: proportion (%) of time in hypoglycemia (<3.9 mmol/L); PT10.0: proportion (%) of time in hyperglycemia (>10 mmol/L). Independent-samples *t*-tests were employed.

**Table 3:** Comparison of the changes in in insulin dosage and clinical features from before to after treatment between the CSII and CSII + Sig groups.

Characteristic	Group		*P* value
	CSII	CSII + Sig	
Δdosage of insulin (U)	4.14 ± 8.59	−2.02 ± 7.50	<0.01
Δbasal insulin dose (U)	1.27 ± 4.59	0.38 ± 4.24	0.44
Δbolus insulin dose (U)	2.87 ± 5.81	−2.40 ± 3.65	<0.01
Δbasal/bolus ratio	0.05 ± 0.89	0.22 ± 0.20	0.31
Δweight (kg)	−0.04 ± 1.38	−0.54 ± 1.18	0.14
ΔBMI (kg/m^2^)	−0.02 ± 0.51	−0.19 ± 0.41	0.15
Time to achieve euglycemia (h)	127.92 ± 27.60	92.88 ± 18.72	<0.01
ΔGA (%)	−4.82 ± 2.75	−8.02 ± 2.90	<0.01
ΔFPG (mmol/L)	−3.85 ± 1.39	−4.39 ± 1.23	0.12
ΔPPG (mmol/L)	−4.20 ± 2.32	−6.45 ± 3.13	<0.01
ΔFC-P (ng/mL)	0.09 ± 0.37	0.21 ± 0.54	0.29
ΔPC-P (ng/mL)	1.46 ± 1.26	1.76 ± 1.57	0.41
ΔCPI	0.63 ± 0.32	0.84 ± 0.42	0.03
ΔSUIT	1.64 ± 0.86	2.20 ± 1.10	0.03
ΔTC (mmol/L)	−0.35 ± 0.40	−0.39 ± 0.65	0.74
ΔHDL-C (mmol/L)	−0.04 ± 0.19	−0.05 ± 0.16	0.82
ΔLDL-C (mmol/L)	−0.12 ± 0.29	−0.22 ± 0.51	0.40
ΔTg (mmol/L)	−0.13 ± 0.20	−0.29 ± 0.50	0.10

Note: Δ: change from before treatment to after. Data are presented as the means ± SD. Paired *t*-tests were employed.
